# Enhancing academic engagement through students’ perceptions of teacher expectations: the mediating role of intentional self-regulation in middle school

**DOI:** 10.3389/fpsyg.2024.1456334

**Published:** 2024-10-24

**Authors:** Haiying Wang, Yueyang Sun, Xin Zhao, Weichen Wang, Jie Xue

**Affiliations:** ^1^School of Psychology, Northeast Normal University, Changchun, China; ^2^Qiaoyi Experimental Middle School, Wuxi, China

**Keywords:** academic engagement, teacher expectation, intentional self-regulation, cross-lagged analyses, adolescent development

## Abstract

In the context of evolving educational standards, enhancing students’ academic engagement has emerged as a critical factor in mitigating the risks of school aversion among middle school students. This study examines the longitudinal effect of middle school students’ perceptions of teacher expectations on their academic engagement, as well as the mediating role of intentional self-regulation in this dynamic. A six-month longitudinal survey was conducted with 702 Chinese middle school students through three waves of questionnaires. The results showed that students’ perception of teacher expectations significantly predicted their academic engagement, with higher perceived teacher expectations leading to increased academic engagement. Furthermore, the study revealed that intentional self-regulation played a pivotal mediating role in the relationship between students’ perceptions of teacher expectations and academic engagement. Students’ perceptions of teacher expectations at Time 1 positively influenced their intentional self-regulation at Time 2, which subsequently enhanced their academic engagement at Time 3. These findings highlight the crucial impact students’ perceptions of teacher expectation on adolescents’ academic motivation and provide guidance for educators to implement proactive strategies that enhance students’ academic development.

## 1 Introduction

Academic engagement plays a vital role in students’ positive academic adjustment, especially during the critical period of early adolescence (Lawson and Lawson, 2013; Wang and Eccles, 2013). It refers to a positive psychological state that individuals maintain throughout their learning process (Schaufeli et al., 2002). This state reflects not only active participation in learning tasks but also integrates aspects of students’ psychological well-being and positive character traits (Wang and Eccles, 2012; Tayama et al., 2019), both of which are fundamental to their overall psychological development. For educators, assessing students’ academic engagement is essential as it significantly predicts their academic achievement, learning satisfaction, and adaptability to school life (Appleton et al., 2006; Li et al., 2022). Furthermore, academic engagement serves as a protective factor by promoting mental health, enhancing well-being, and reducing learning burnout and dropout rates (Carmona-Halty et al., 2021; Abreu Alves et al., 2022).

In China’s fundamental education system, the middle school stage represents a critical transitional period marked by a significant escalation in academic difficulty compared to elementary school. Consequently, the issue of behavioral disengagement among middle school students has become an urgent problem to address. In this context, research on academic engagement is of considerable significance. With the rise of positive psychology, more researchers are focusing on student academic engagement as a constructive state to combat school refusal behavior (Solomon and Croft, 2016; Abreu Alves et al., 2022; Widlund et al., 2023).

Middle school adolescents are at a critical juncture of both educational and psychological growth. Their academic engagement is largely shaped by the surrounding environment and significant individuals who influence their academic experiences (Carmona-Halty et al., 2021; McKellar and Wang, 2023). Within this context, teachers emerge as pivotal figures in the school setting, having a lasting and profound influence on students’ attitudes and behaviors toward learning (Goetz et al., 2021). Over half a century ago, Rosenthal and Jacobson (1968) concluded that a teacher’s belief in and expectations for a student’s potential could lead to self-fulfilling prophecies. Conversely, low expectations from teachers can negatively impact students’ motivation by limiting learning opportunities and creating a negative learning environment (Weinstein, 2002). It is important to recognize that the impact of teacher expectations is primarily realized through students’ perceptions. Once these perceived expectations are internalized, they significantly influence students’ motivation and academic performance (Johnston et al., 2023). This underscores the necessity of focusing on how students interpret and internalize teacher expectations in educational research and practice.

Meanwhile, the internal factors also play an important role in the interplay between their perception of teacher expectations and academic engagement. Meanwhile, students’ internal factors can play an important role in the interplay between their perception of teacher expectations and academic engagement. Grounded in the situated expectancy-value theoretical framework (Wigfield and Eccles, 2000; Eccles and Wigfield, 2020), intentional self-regulation can be viewed as a comprehensive process in which students re-evaluate and adjust their academic-related cognitive structures and expectations, and adopt corresponding adjustment strategies after perceiving the beliefs and behaviors of others (Eccles and Wigfield, 2020; Gestsdóttir and Lerner, 2008; Zhong et al., 2022). This process, in turn, drives their subsequent academic outcomes. Longitudinal research has also shown that intentional self-regulation can positively impact student academic engagement over time (Stefansson et al., 2018). Thus, when students perceive and internalize their teachers’ expectations, their self-regulation may lead to the adjustment of short-term learning goals, influencing their subsequent academic engagement.

To gain a more in-depth understanding of these dynamic influences, this study focuses on middle school students, employing a three-wave longitudinal survey to explore the impact of teacher expectations on their academic engagement from the students’ perspective and the role of intentional self-regulation in this process.

### 1.1 Students’ perceptions of teacher expectations and academic engagement

In recent years, the effect of teacher expectations on student academic engagement has received significant attention in the field of educational psychology (Wang et al., 2018; Neuenschwander et al., 2021). Teachers’ beliefs and attitudes play an important role in determining students’ motivation and their learning behaviors. This influence can manifest through explicit expressions, subtle behaviors, and feedback, with students typically adjusting their learning behaviors in response to these cues (Jussim and Harber, 2005; Rubie-Davies et al., 2020; Hornstra et al., 2023). Rosenthal and Jacobson’s (1968) study first identified that teacher expectations can significantly elevate student performance, a phenomenon later known as the “Pygmalion effect.” Subsequent studies have delineated the mechanisms of this effect, demonstrating the nuanced ways in which teacher expectations enhance student learning outcomes. Despite criticisms of these experimental methods, researchers have consistently acknowledged the existence of teacher expectation effects (Gentrup et al., 2020; Hollenstein et al., 2024). For instance, Hornstra et al. (2018) argued that one prerequisite for forming learning motivation is the perception of positive expectations from teachers, which in turn affects their engagement in learning. Another study found that teachers who hold high expectations are inclined to provide more challenging tasks, give positive feedback regularly, and directly enhance student academic engagement and motivation (Rubie-Davies et al., 2012). These teachers offer more choices in learning activities, apply teaching methods that foster motivation, track students’ educational progress continuously, and encourage independent learning (Rubie-Davies et al., 2006; Rubie-Davies, 2007; Hornstra et al., 2018; Johnston et al., 2022). Conversely, teachers with low expectations are more likely to use direct instruction, offer limited choices to students, provide less feedback, and tend to group students rigidly by ability. Such practices may lead to a decline in students’ interest in learning and a decrease in adopting behaviors that enhance learning (Rubie-Davies, 2007; Johnston et al., 2022).

Drawing on the concept of the teacher expectation effect, existing research has illuminated the significant impact that both actual and perceived teacher expectations can exert on students’ psychological well-being and academic performance (Wang et al., 2018). Nonetheless, it is important to acknowledge that the subjective expectations held by teachers do not spontaneously engender self-fulfillment in students (Rubach et al., 2023). Teacher expectations can subtly permeate classroom interactions, manifesting in spoken words, nonverbal gestures, vocal inflections, and feedback on student assignments (Johnston et al., 2023). As certain researchers have pointed out, the activation of the teacher expectation effect is predicated upon the students’ recognition and interpretation of these communicated signals (Weinstein, 2002; Johnston et al., 2023; Rubach et al., 2023). Although students are the primary subjects affected by these expectations, much of the research has predominantly been through the lens of teachers and other professionals in the field. Only a few recent studies have examined the actual impact of teacher expectations from the students’ own perspective (Hornstra et al., 2018; Rubie-Davies et al., 2020; Johnston et al., 2023). In reality, the impact of teacher behavior on students’ learning largely depends on how students perceive those behaviors, which may not always align with the teachers’ intentions (Johnston et al., 2023). Studies have also shown that students modulate their academic behaviors in response to the expectations they discern from teachers, aiming to fulfill these anticipations and receive positive feedback (Lavy and Naama-Ghanayim, 2020). When students perceive strong signals of expectations, they tend to experience increased support from their teachers, leading to improved academic performance (Gaspard et al., 2023; Johnston et al., 2023). Therefore, this study aimed to explore teacher expectations from students’ perspective, examining the impact of these perceived expectations on student academic engagement. In this study, students’ perception of teacher expectations are defined as their interpretation of predictive beliefs about their academic achievement inferred from teachers’ everyday actions and attitudes.

Moreover, students’ perceptions of teachers expectations may vary according to their achievements and developmental progress (Hughes and Kwok, 2006; Timmermans et al., 2021; Hao et al., 2022). This dynamic suggests that the academic engagement and successes of students may conversely shape their perceptions of teacher expectations, thus fostering a reciprocal relationship. Nevertheless, scant research has delved into the dynamics or causal relationships between perceived teacher expectations and academic engagement (Kelly and Carbonaro, 2012; Timmermans et al., 2021), with the existing studies primarily utilizing cross-sectional approaches that captured a momentary, static correlation (An et al., 2023). Therefore, this research employed a longitudinal approach to investigate the relationship between student’s perception of teacher expectations and academic engagement among middle school students, aiming to provide robust theoretical support and practical guidance for educational practice.

### 1.2 The mediating role of intentional self-regulation

The situated expectancy-value theory provides a comprehensive framework for understanding how students’ perceptions of teacher expectations can impact their academic engagement (Eccles and Wigfield, 2020). This model proposes a sequential process: students’ perceptions of others beliefs and behaviors affect their general self-schemata, leading to changes in their expectations of success, and ultimately impacting achievement-related choices and performance (Wigfield and Eccles, 2000; Eccles and Wigfield, 2020). Within this context, perceived teacher expectations constitute a significant external factor, shaping students’ academic self-schemata, learning goals, and expectations for success, positively affecting their academic engagement (Diemer et al., 2016; Han et al., 2022; Rubach et al., 2023).

From a dynamic perspective, the process of intentional self-regulation involves students adjusting their cognition and behavior to coordinate their perceived teacher expectations with their own academic goals. This comprehensive process, spanning from students modifying their general self-schemata to establishing specific academic goals and success expectations, essentially reflects the process of students’ intentional self-regulation. Intentional self-regulation encompasses the active management of behaviors, emotions, and cognitions in pursuit of personal goals (Gestsdóttir and Lerner, 2008; Zimmerman et al., 2008). It is particularly crucial during adolescence, a critical period for self-regulation development, where adolescents must proactively apply strategies to balance their needs and resources in different situations (Gestsdóttir and Lerner, 2008; Tian et al., 2015). Freund and Baltes (2002) proposed the SOC model based on the nature of intentional self-regulation functions during adolescence. It posits that students can allocate resources or adjust their learning behaviors to align with their goals and competencies by employing three strategies: Selection (S), Optimization (O), and Compensation (C) (Gestsdóttir and Lerner, 2007; Moghimi et al., 2017). Studies have shown that intentional self-regulation is not only highly related to students’ academic engagement (Larson and Tran, 2014; Stefansson et al., 2018) but also significantly enhances their attentiveness to educational activities (Gestsdóttir et al., 2023). This, in turn, contributes to a deeper level of involvement in their learning process (Larson and Tran, 2014; Zhou et al., 2021; Gestsdóttir et al., 2023). Therefore, based on the situated expectancy-value theoretical framework, this study conceptualizes intentional self-regulation as a comprehensive process ranging from goals and general self-schemata to the formation of success expectations. Through this dynamic and active self-regulation, students can continuously adjust their internal cognition and motivation by integrating external resources, thus forming a constantly updated dynamic relationship between teacher expectations and academic engagement.

Existing research has illuminated that intentional self-regulation bridges the enduring relationship between internal and external learning resources (Gestsdóttir et al., 2011; Chang et al., 2020). This implies that students’ perceptions of teacher expectations, seen as an external resource, and student academic engagement, an internal resource, are interconnected not just through direct association but also via an indirect pathway by shaping intentional self-regulation (Xu et al., 2017). Intentional self-regulation can be regarded as an evolving variable responsive to alterations in environmental contexts. The study carried out by Bowers et al. (2016) has shown that mentoring relationships can affect adolescents’ intentional self-regulation. Given their pivotal role in students’ lives, perceived teacher expectations can directly influence or indirectly shape student behavior through the mediation of the students’ own self-expectations. A longitudinal study of Asian-American high school students in the United States revealed that higher perceived teacher expectations positively correlated with students’ success expectations and academic achievements (Cherng and Liu, 2017). Moreover, Intervention and interview studies have also indicated that students who have a clear understanding of their teachers’ expectations demonstrate improved intentional self-regulatory behaviors (Vattøy, 2020; Wahman and Anderson, 2021).

Grounded in both theoretical frameworks and empirical findings, there appear to be pairwise correlations among students’ perceived teacher expectations, intentional self-regulation, and academic engagement. Thus, the longitudinal relationship between students’ perceptions of teacher expectations and student academic engagement is likely mediated by intentional self-regulation. In other words, students’ perceptions of teacher expectations initially affect students’ intentional self-regulation, which in turn impacts their academic engagement (Larson and Tran, 2014; Stefansson et al., 2018; Xu et al., 2017; Yerdelen and Sungur, 2019). Furthermore, the variables of students’ perceptions of teacher expectations, self-regulation, and academic engagement in middle school are inherently dynamic, adapting progressively in concert with environmental shifts (Bowers et al., 2016; Quin, 2017; Vattøy, 2020; Wahman and Anderson, 2021). Hence, this study posits that intentional self-regulation could serve as a mediator between middle school students’ perceptions of teacher expectations and academic engagement, fostering a dynamic interplay over time.

### 1.3 The present study

Although perceived teacher expectations act as a critical catalyst for middle school students’ academic engagement, uncovering the mechanisms by which students leverage this catalyst remains paramount. Prior research has explored the association between students’ perceptions of teacher expectations and academic engagement (Yerdelen and Sungur, 2019; Lavy and Naama-Ghanayim, 2020), and has begun to consider cognitive factors in internalizing these expectations (Cherng and Liu, 2017; Quin, 2017). However, a nuanced understanding remains to be developed regarding how students engage in intentional self-regulation to navigate the influence of internalized perceived teacher expectations on their academic engagement. To further elucidate this complex relationship, the present study integrated the concept of intentional self-regulation into the expectancy-value theoretical framework to examine its impact on the interplay between teacher expectations and the academic engagement of middle school students.

**Figure 1:**
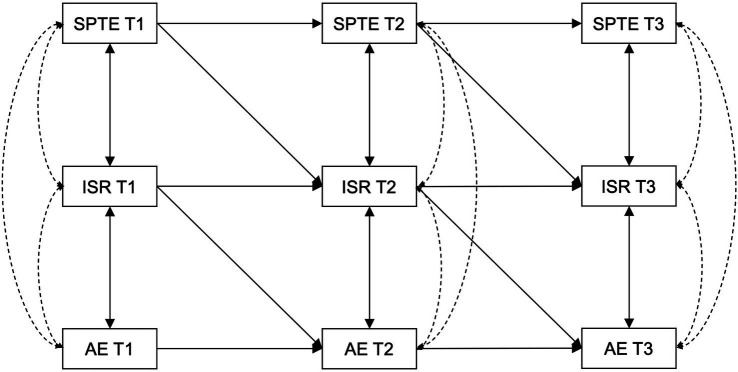
Hypothesized mediated cross-lagged model. SPTE, students’ perceptions of teacher expectations; ISR, intentional self-regulation; AE, academic engagement; T1, T2, T3, Time 1, Time 2, Time 3.

Teachers’ expectations of their students are adaptable and subject to alterations driven by external factors. Concurrently, students are gradually aware form perceptions of these expectations and modify their behavior in response, constituting an ongoing interaction between teachers and students (Cherng and Liu, 2017; Quin, 2017). Intentional self-regulation serves as a pivotal adjustment mechanism that enables students to swiftly adapt to the changes in their perceptions of teacher expectations, affecting their academic engagement. Consequently, this investigation employed cross-lagged panel modeling techniques to examine the relationship between students’ perceptions of teacher expectations and student academic engagement within a middle school setting. Furthermore, it scrutinized the mediation effect of intentional self-regulation on the interplay between these variables through longitudinal mediation analysis. The conceptual model, depicted in Figure 1, posits two primary hypotheses: (1) Students’ perceptions of teacher expectations exert a positive influence on academic engagement; and (2) Intentional self-regulation acts as a longitudinal mediator in the relationship between students’ perceptions of teacher expectations and academic engagement.

## 2 Materials and methods

### 2.1 Participants and procedure

The present study was conducted employing a longitudinal design over a six-month period, involving 788 middle school students sourced from three educational institutions within Jilin Province, selected through convenience sampling methodology. Data collection ensued at three distinct junctures, each demarcated by a three-month interval. This procedure encompassed the administration of three sequential waves of questionnaire surveys to the participants: the initial wave at the beginning of the spring semester (Time 1), followed by a subsequent wave at the end of the spring semester (Time 2), and the final wave at the beginning of the fall semester (Time 3). Considering Chinese middle schools include grades 7 to 9, and ninth graders graduating at the end of the spring semester, our study focused exclusively on seventh and eighth graders from Time 1.

In the matching and screening process of the three questionnaire datasets, we initially omitted students who were unable to continue due to illness, transfer, or dropout. To protect privacy and improve the survey quality, an anonymous approach was adopted, collecting only essential identifiers: students’ school identification numbers, grades, classes, age, and gender. This information facilitated the precise matching of each participant’s responses across questionnaires. The preliminary screening focused on the duration of response time and the completeness of the questionnaires. The R package careless was utilized to detect instances of inattentive responses, yielding a final effective sample size of 702 individuals. This reflects a total attrition rate of 10.91% by Time 3. Within this valid sample, there were 313 males (44.59%) and 389 females (55.41%), comprising 387 seventh graders (55.13%) and 315 eighth graders (44.87%). The age of participants ranged from 12 to 16 years, with a mean age of 14.18 ± 0.91.

An independent sample t-test was implemented to assess biases arising from participant attrition. Specifically, participants who completed all three questionnaires were coded as 1, and those with incomplete responses were coded as 0. The test results indicated no statistically significant disparities in academic engagement (*t* = −0.36, *p* > 0.05), teacher expectation (*t* = 1.24, *p* > 0.05), or intentional self-regulation (*t* = −0.24, *p* > 0.05) between the groups with complete and incomplete datasets.

The study was executed within classroom settings, utilizing paper-based questionnaires. Administration was conducted by psychology master’s students who had undergone specialized training to fully comprehend the testing procedures and requirements before the survey deployment. The instruments used for all three waves were consistent. The study adhered to the principles laid out by the research ethics committee and received approval from the principals of the participating schools. Participants were informed about the study purpose, the voluntary basis of their participation, and their right to withdraw from the survey at any time. Informed consent was obtained from all participants and their guardians prior to their inclusion in the study.

### 2.2 Measures

Data collection in this study was based on self-reported assessments. Although Cronbach’s alpha (*α*) has long been the standard for reliability assessment, recent literature advocates for McDonald’s omega (*ω*) as a more robust measure of internal consistency (Hayes and Coutts, 2020). In alignment with this contemporary viewpoint, this study utilized Jamovi 2.2.5 to compute the McDonald’s *ω* coefficient, thereby serving as the reliability index for adopted questionnaires. Confirmatory factor analysis (CFA) was performed using Mplus 8.3 to verify the structural validity of the survey instruments.

#### 2.2.1 Students’ perceptions of teacher expectations questionnaire

The present study employed Zhang’s (2009) Student Perception of Teacher Expectancy Questionnaire, a robust tool featuring the response options on a Likert scale, ranging from 1 (“strongly disagree”) to 5 (“strongly agree”). The questionnaire encompasses 15 items across three dimensions: teacher support (e.g., “The teacher always cares about me”), instructional interaction (e.g., “The teacher held me to higher academic standards than my other classmates”), and academic feedback (e.g., “When I ask the teacher for help, he/she usually explains things to me patiently”), with aggregate scoring reflecting the intensity of a student’s perception of teacher expectations. The questionnaire was specifically tailored to align with the Chinese educational framework, distinguished by its classroom-based instruction approach, wherein homeroom teachers play a crucial role in overseeing the students’ comprehensive academic performance. Reflecting this emphasis, the current study specifically targeted the perceived expectations held by homeroom teachers. The questionnaire demonstrated high internal consistency across three measurement waves, with McDonald’s *ω* coefficients of 0.92, 0.91, and 0.91, respectively. Confirmatory factor analysis (CFA) yielded good fit indices, with *χ^2^/df* ranging from 3.08 to 3.58, CFI between 0.96 and 0.97, TLI from 0.95 to 0.96, and RMSEA consistently at 0.06, indicating a well-fitting model for the questionnaire.

#### 2.2.2 Intentional self-regulation index

The Adolescent Intentional Self-Regulation Index, created by Gestsdóttir and Lerner (2007), was used to measure participants’ intentional self-regulation. The questionnaire consists of 9 items across three dimensions: goal selection (e.g., “When I decide upon a goal, I stick to it.”), goal optimization (e.g., “I keep trying as many different possibilities as are necessary to succeed at my goal”), and goal compensation (e.g., “For important things, I pay attention to whether I need to devote more time or effort”). Participants rated these items on a 7-point Likert scale (1 = “strongly disagree,” 7 = “strongly agree”), with higher total scores indicating a higher level of intentional self-regulation. The McDonald’s ω coefficients for the questionnaire across three measurements were 0.89, 0.90, and 0.89. The confirmatory factor analysis (CFA) fit indices were favorable, with *χ^2^/df* ranging from 2.66 to 4.74, CFI from 0.97 to 0.99, TLI from 0.96 to 0.99, and RMSEA from 0.05 to 0.07.

#### 2.2.3 Academic engagement scale

Student academic engagement was measured using The Utrecht Work Engagement Scale for Students (Schaufeli et al., 2002). The scale consists of 17 items that are divided into three dimensions: vigor (e.g., “When I get up in the morning, I feel like going to class”), dedication (e.g., “I am enthusiastic about my studies”), and absorption (e.g., “When I am studying, I forget everything else around me”). Participants responded to this scale on a 7-point Likert scale, ranging from 1 (“never”) to 7 (“always”), where a higher cumulative score indicated a greater level of academic engagement. The scale demonstrated high reliability, with McDonald’s ω coefficients consistently at 0.93 across three measurements. Confirmatory factor analysis (CFA) showed good fit indices, with *χ^2^/df* between 3.20 and 3.38, CFI ranging from 0.96 to 0.97, TLI at 0.96, and RMSEA at 0.06, indicating a well-fitting model for the questionnaire.

### 2.3 Data processing

The study utilized SPSS 26.0 and Mplus 8.3 for data analysis. The initial step involved conducting descriptive statistics and computing correlation coefficients to assess the stability and interrelationships of students’ perceptions of teacher expectations, intentional self-regulation, and academic engagement across different measurement points. This preliminary analysis aimed to identify any significant trends or consistencies within the data, establishing the foundation for further in-depth analysis.

Informed by the correlational analysis findings, a cross-lagged model was developed to explore the relationships between teacher expectations and academic engagement over time. This step examined the directionality and strength of the relationships. The model fitting process, which included examining the fit indices, was executed in Mplus 8.3 to ascertain the model’s suitability and better capture the underlying dynamics.

After confirming the validity of the model, a longitudinal mediation analysis was implemented to elucidate the pathways through which students’ perceptions of teacher expectations influence their academic engagement, specifically focusing on the mediating role of intentional self-regulation. The mediation analysis provided a comprehensive understanding of the indirect effects, thereby providing insights into the intricate interplay of the studied variables.

## 3 Results

### 3.1 Common method bias test

Given that all variable measurements in this study were based on self-reports by subjects, there was a potential risk of common method bias. To assess this potential bias, we employed Harman’s single-factor test (Podsakoff et al., 2012). The results of the exploratory factor analysis indicated that the variance explained by the principal factor at Time 1 (T1), Time 2 (T2), and Time 3 (T3) was 30.41, 31.45, and 25.06%, respectively. Each percentage was below the 40% critical threshold, which suggested that common method bias was not a significant concern in this study.

### 3.2 Descriptive statistics and correlational analysis

The results of descriptive statistics, correlational analyses, t-tests, and ANOVA for all variables in the study are presented in Table 1. Significant positive correlations were observed among students’ perceptions of teacher expectations, intentional self-regulation, and academic engagement at each of the three measurement intervals, indicating the temporal stability of these variables within the middle school population. Within the same measurement points, significant positive relationships were consistently observed between students’ perceptions of teacher expectations, intentional self-regulation, and academic engagement. Moreover, across different measurement points, students’ perceptions of teacher expectations and intentional self-regulation at T1 exhibited a significant positive correlation with academic engagement at T2. Similarly, students’ perceptions of teacher expectations and intentional self-regulation at T2 were also significantly associated with academic engagement at T3. Analysis of variance (ANOVA) results indicated no significant differences attributable to age across the variables. Likewise, t-test analyses confirmed the absence of significant gender disparities across all measured variables.

**Table 1:** Descriptive statistics, analysis of variance, *T*-test, and correlation analysis for the variables.

Variables	M ± SD	*F*	*t*	1	2	3	4	5	6	7	8
1 SPTE (T1)	3.55 ± 0.94	0.676	−1.470	1							
2 SPTE (T2)	3.56 ± 0.78	1.781	−0.475	0.478^**^	1						
3 SPTE (T3)	3.60 ± 0.74	1.038	0.623	0.333^**^	0.297^**^	1					
4 ISR (T1)	4.45 ± 1.58	0.737	1.952	0.423^**^	0.265^**^	0.135^**^	1				
5 ISR (T2)	4.51 ± 1.29	1.045	−0.546	0.617^**^	0.526^**^	0.283^**^	0.426^**^	1			
6 ISR (T3)	4.54 ± 1.17	1.202	0.909	0.398^**^	0.476^**^	0.320^**^	0.359^**^	0.477^**^	1		
7 AE (T1)	4.64 ± 1.40	1.346	−1.533	0.304^**^	0.233^**^	0.149^**^	0.262^**^	0.263^**^	0.192^**^	1	
8 AE (T2)	4.45 ± 1.34	0.949	−0.488	0.200^**^	0.348^**^	0.103^**^	0.412^**^	0.359^**^	0.302^**^	0.285^**^	1
9 AE (T3)	4.34 ± 0.91	1.712	−0.614	0.497^**^	0.432^**^	0.423^**^	0.367^**^	0.600^**^	0.535^**^	0.358^**^	0.393^**^

The *F* value denotes the results of the age variance analysis, and the t value signifies the outcomes of gender different test; **p < 0.01. SPTE, Students’ Perceptions of Teacher Expectation; ISR, Intentional Self-regulation; AE, Academic Engagement; T1, T2, T3, Time 1, 2, and 3, respectively.

### 3.3 Cross-lagged analysis between students’ perceptions of teacher expectations and academic engagement

**Figure 2:**
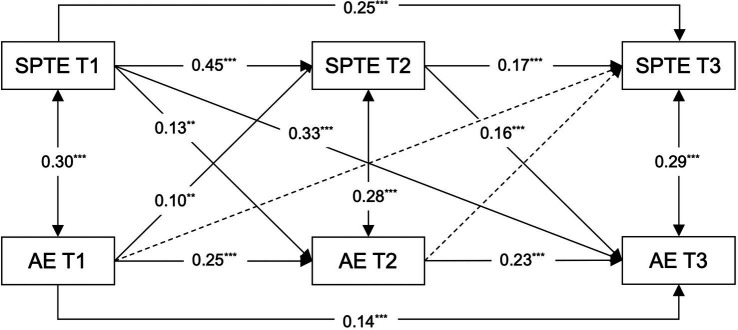
Path coefficients in Model 1. SPTE, students’ perceptions of teacher expectations; ISR, intentional self-regulation; AE, academic engagement; T1, T2, T3, Time 1, Time 2, Time 3. The straight line represents a significant path, and the dashed line represents an insignificant path. All regression coefficients are standardized and labeled as significant in the figure, with **p < 0.01, ***p < 0.001.

Building on the correlational analyses and controlling for age, this study constructed a cross-lagged model between students’ perceptions of teacher expectations and academic engagement across three waves (Model 1, see Figure 2). The model exhibited fit indices of *χ^2^/df* = 3.90, RMSEA = 0.06, CFI = 0.99, and TLI = 0.95, demonstrating a satisfactory fit, thereby justifying the acceptance of the model’s results (see Table 2). Figure 2 illustrates the regression coefficients for each pathway.

**Table 2:** Fit indices for cross-lagged panel models.

	*χ^2^*	*df*	CFI	TLI	RMSEA	SRMR
Model 1	3.895	1	0.996	0.950	0.064	0.013
Model 2	30.842	13	0.990	0.975	0.044	0.033
Model 3	642.625	13	0.648	0.106	0.263	0.167
Model 4	16.185	8	0.995	0.981	0.038	0.018

In the cross-lagged analysis examining the relationship between students’ perceptions of teacher expectations and academic engagement, students’ perceptions of teacher expectations at T1 and T2 were found to positively predict academic engagement at subsequent time points. Conversely, while students’ academic engagement at T1 positively predicted their perceived teacher expectations at T2, academic engagement at T2 did not significantly influence students’ perceptions of teacher expectations at T3. Notably, students’ perceptions of teacher expectations at T1 were significant predictors of academic engagement at T3. In addition, students’ perceptions of teacher expectations at T1 were found to predict students’ perceptions of teacher expectations at T3, and academic engagement at T1 predicted academic engagement at T3. These results indicated a delayed predictive effect of middle school students’ perceptions of teacher expectations on academic engagement, and highlighted cross-temporal stability for both constructs. The findings thus validate Hypothesis 1 and set the stage for subsequent longitudinal mediation effect analysis.

### 3.4 Longitudinal analysis of intentional self-regulation as a mediator

**Figure 3:**
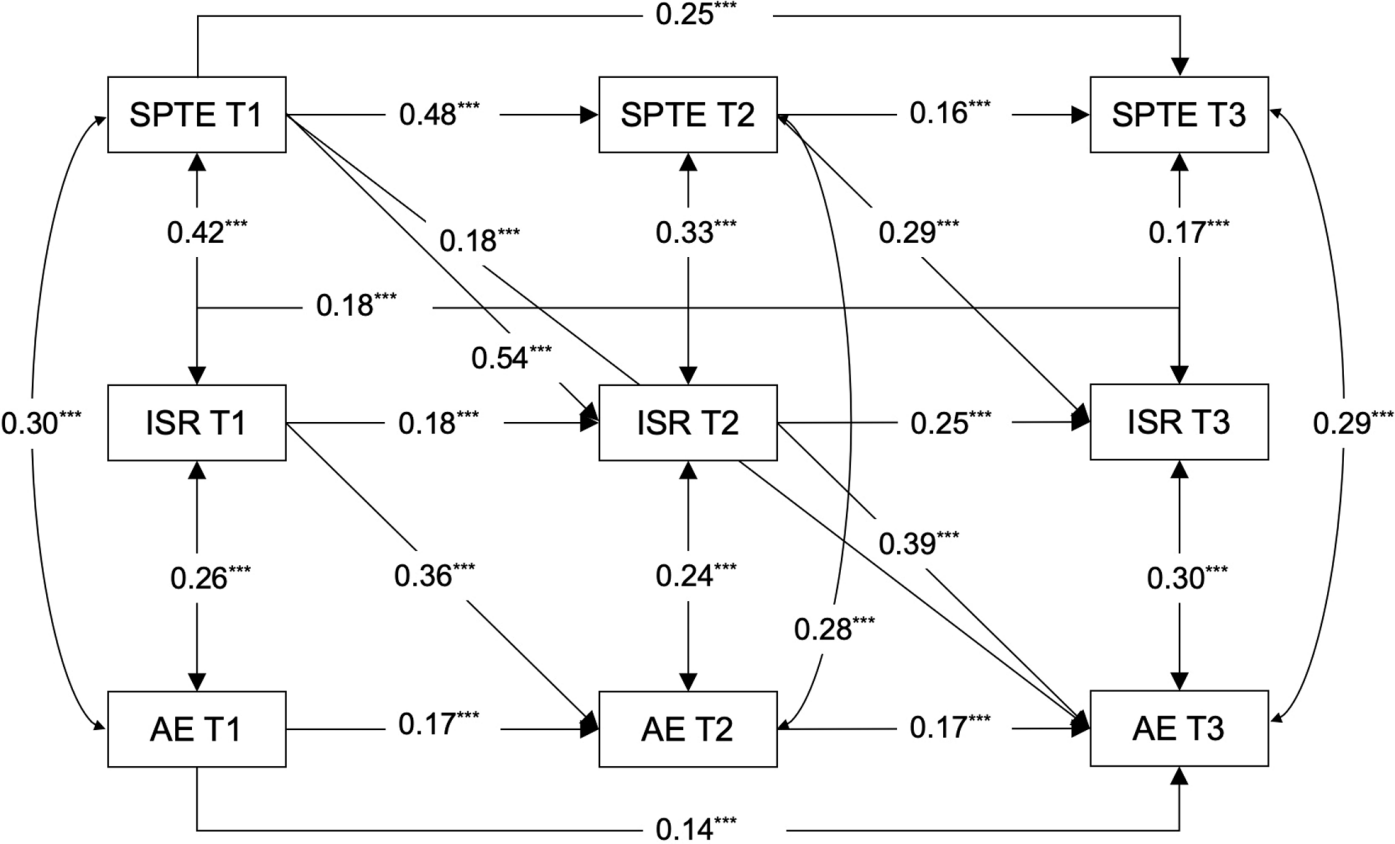
Path coefficients in Model 2. SPTE, students’ perceptions of teacher expectations; ISR, intentional self-regulation; AE, academic engagement; T1, T2, T3, Time 1, Time 2, Time 3. The straight line represents a significant path, and the dashed line represents an insignificant path. All regression coefficients are standardized and labeled as significant in the figure, with ***p < 0.001.

**Figure 4:**
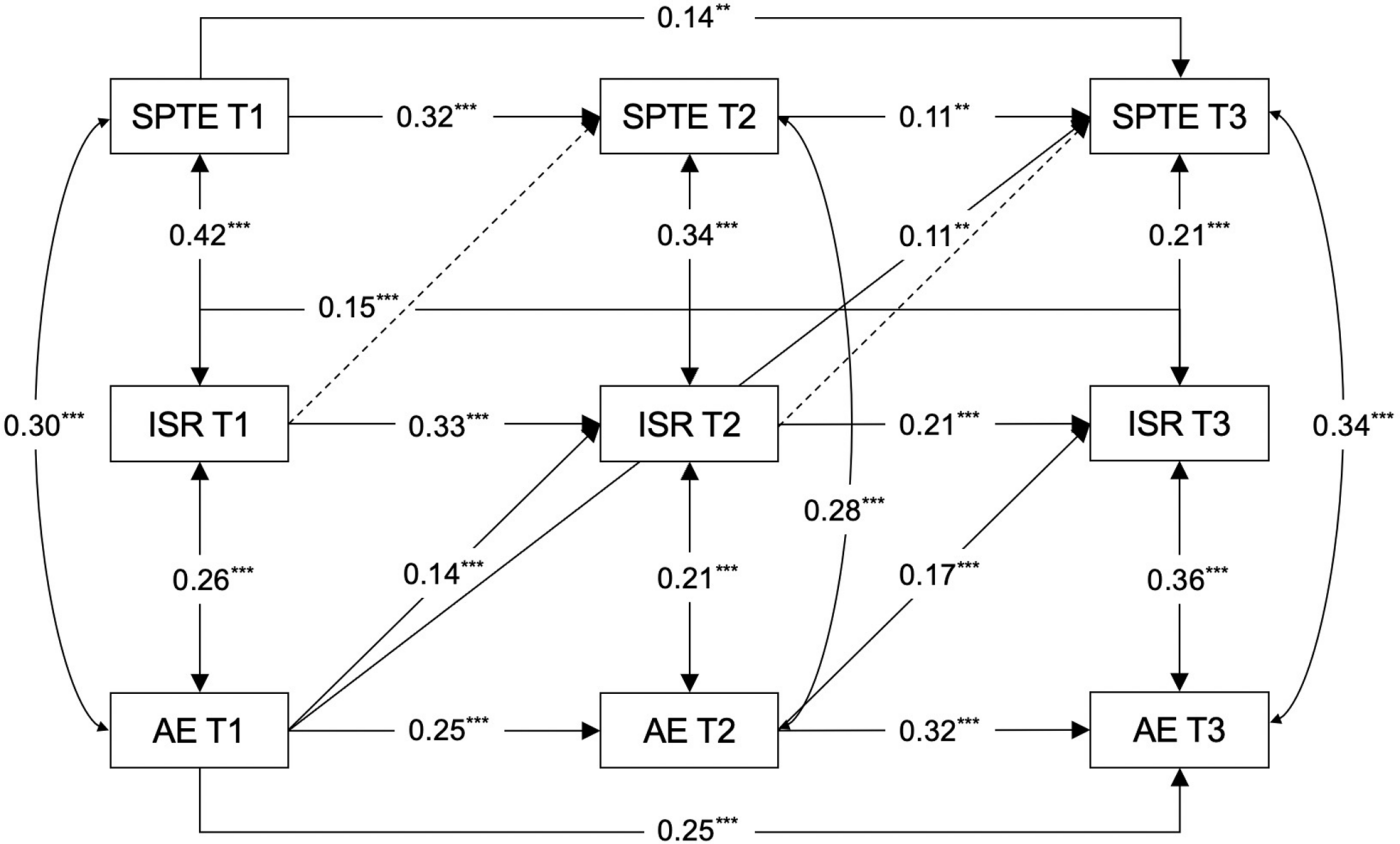
Path coefficients in Model 3. SPTE, students’ perceptions of teacher expectations; ISR, intentional self-regulation; AE, academic engagement; T1, T2, T3, Time 1, Time 2, Time 3. The straight line represents a significant path, and the dashed line represents an insignificant path. All regression coefficients are standardized and labeled as significant in the figure, with **p < 0.01, ***p < 0.001.

**Figure 5:**
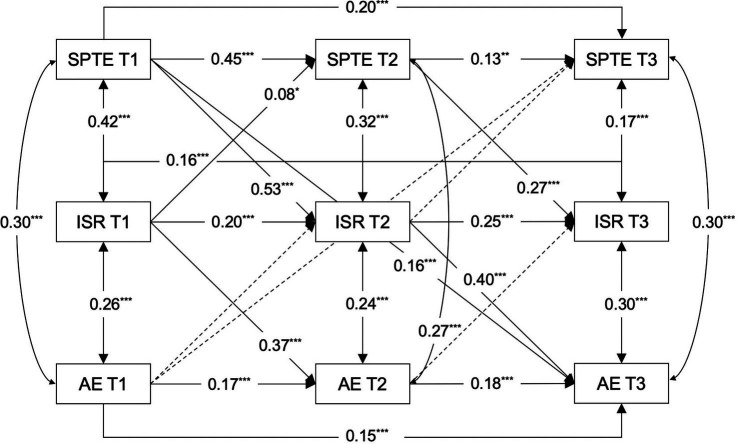
Path coefficients in Model 4. SPTE, students’ perceptions of teacher expectations; ISR, intentional self-regulation; AE, academic engagement; T1, T2, T3, Time 1, Time 2, Time 3. The straight line represents a significant path, and the dashed line represents an insignificant path. All regression coefficients are standardized and labeled as significant in the figure, with *p < 0.05, **p < 0.01, ***p < 0.001.

Expanding upon the findings from the cross-lagged analysis between students’ perceptions of teacher expectations and academic engagement, and incorporating age as a covariate, this investigation developed three distinct models to examine the bidirectional mediation of intentional self-regulation in the relationship between students’ perceptions of teacher expectations and academic engagement. The first model probed the process by which students’ perceptions of teacher expectations might affect their academic engagement through intentional self-regulation (Model 2, see Figure 3). The second model explored the reverse pathway, analyzing how student academic engagement could influence their perceptions of teacher expectations through intentional self-regulation (Model 3, see Figure 4). The third model encompassed all potential cross-lagged paths among the studied variables (Model 4, see Figure 5). As shown in Table 2, both Models 2 and 4 demonstrated a good fit, whereas Model 3 did not align well with the empirical data. A comparative analysis of the *χ^2^* values for Models 2 and 4 indicated a superior fit for Model 2 (∆*χ^2^* = 14.297, ∆*df* = 5, *p* < 0.05). Therefore, Model 2 was selected as the final mediation model for further cross-lagged analysis.

The results revealed significant pathways within the mediation model: Students’ perceptions of teacher expectations at T1 positively predicted intentional self-regulation at T2 (*β* = 0.54, *p* < 0.001), and similarly, students’ perceptions of teacher expectations at T2 positively influenced intentional self-regulation at T3 (*β* = 0.29, *p* < 0.001). In the subsequent phase of the mediation pathway, intentional self-regulation at T1 positively predicted academic engagement at T2 (*β* = 0.36, *p* < 0.001), and intentional self-regulation at T2 positively predicted academic engagement at T3 (*β* = 0.39, *p* < 0.001).

Intentional self-regulation significantly mediated the relationship between students’ perceptions of teacher expectations and academic engagement. This mediation effect was evidenced by a significant indirect effect of 0.212 and a 95% confidence interval of [0.156, 0.252] (not including 0, *p* < 0.001), accounting for 54.78% of the total effect. This finding highlight the important role of intentional self-regulation in the relationship between students’ perceptions of teacher expectations and academic engagement, in line with Hypothesis 2. Table 3 details the direct, indirect, and total effects within the established mediation model (Model 2).

**Table 3:** Path coefficient analysis of mediation model.

Path	Effect	SE	95%CI	
			LLCI	ULCI
Total effect	0.387	0.033	0.292	0.438
Specific indirect	0.212	0.023	0.156	0.252
Direct	0.175	0.039	0.063	0.237

## 4 Discussion

This study aimed to explore the relationship between students’ perceptions of teacher expectations and academic engagement among middle school students, examining the mediating role of intentional self-regulation. Utilizing data collected over three waves and employing cross-lagged and longitudinal mediation analyses, our findings illuminate the predictive effect of students’ perception of teacher expectations on students’ academic engagement. Significantly, this research highlights the mediating role of intentional self-regulation, emphasizing its importance in the interaction between students’ perception of teacher expectations and academic engagement. Furthermore, our analysis revealed a unidirectional influence from students’ perceptions of teacher expectations to academic engagement, confirming the significant role of perceived teacher attitudes and behaviors in shaping student outcomes. Overall, these findings not only demonstrated a clear temporal progression but also affirmed a significant relationship among the studied variables, deepening our comprehension of how students’ perceptions of teacher expectations relate to student academic outcomes over time.

### 4.1 The impact of students’ perceptions of teacher expectations on academic engagement

This study employed a cross-lagged analysis to investigate the interactive relationship between students’ perceptions of teacher expectations and their academic engagement. The results revealed a significant positive predictive relationship between middle school students’ perceptions of teacher expectations and their subsequent academic engagement. This finding aligns with and extends previous research across intervention, interview, and cross-sectional studies (An et al., 2023; Gaspard et al., 2023; Johnston et al., 2023), thereby supporting Hypothesis 1. Our longitudinal approach further offered compelling evidence of the directional influence of students’ perceptions of teacher expectations on academic engagement over time, emphasizing the enduring importance of positive teacher expectations.

Specifically, students who reported perceiving higher expectations from their teachers also demonstrated higher levels of academic engagement. While our study focused on students’ perceived expectations rather than directly measured teacher expectations, this observed trend aligns with the core principles of teacher expectation effects in the context of academic engagement. The similarity in the direction of influence—from teacher expectations (in our case, students’ perceptions of teacher expectations) to student outcomes—parallels the fundamental mechanism proposed by the Pygmalion effect, albeit with our specific focus on students’ perceptions. This finding enriches the discourse established by various research methodologies, underscoring the importance of sustained, positive teacher expectations in a broader view, showcasing their pivotal role not just in immediate student responses, but in shaping academic engagement trajectories over more extended periods.

Academic engagement, reflecting the degree of a student’s absorption, vigor, and dedication in the learning process (Schaufeli et al., 2002), was examined as an outcome potentially influenced by students’ perceptions of teacher expectations. Consistent with prior research, our study underscored that these perceptions are instrumental in positively affecting student academic engagement (Johnston et al., 2022). Crucially, the influence of teacher expectations on students largely depends on how students perceive these expectations (Rubach et al., 2023).

Teachers express their expectations through specific actions, including academic guidance, learning support, and attention to students’ attitudes and performance. The emotional support inherent in teachers’ expectations becomes a critical factor in this process. Research by Yin et al. (2023) illustrated that the emotional components, such as love, trust, and respect, inherent in teacher expectations collectively foster a nurturing teacher-student relationship. This relationship is fundamental, as students who perceive these positive emotions are more likely to adopt proactive behaviors and attitudes toward learning. Such perceptions inspire them to elevate their academic standards and enhance their engagement in learning activities.

Moreover, middle school students are in a pivotal phase where external evaluations significantly influence their self-esteem (Dan et al., 2014; Gong et al., 2023). In this context, teachers play a crucial role in shaping their students’ academic experiences. Teachers’ high expectations and emotional investment, evident in heightened classroom challenges, extended problem-solving opportunities, and enriched teacher-student interactions, can persistently stimulate and unlock students’ potential. When students perceive and internalize these high expectations and supportive practices form their teachers, they become inherently motivated to confront challenges, engage in active exploration, and tailor personalized learning strategies. These positive perceptions of teacher expectations significantly enhances their overall learning experience (An et al., 2023).

Our study demonstrated the significant impact of middle school students’ perceptions of teacher expectations on their academic engagement, a finding complemented by Reeve et al.'s (2019) research showing that enhancing teachers’ motivating styles can improve these perceptions and, consequently, increase engagement. These findings align well with the situated expectancy-value theory (Wigfield and Eccles, 2000; Eccles and Wigfield, 2020), which posits that students’ perceptions of their social environment, including teacher expectations, shape their academic beliefs and outcomes. However, the relationship between perceived teacher expectations and academic engagement is not likely to be direct. Rather, it may operate through various internal processes that students undergo as they interpret and respond to their perceived environment. In the situated expectancy-value theoretical framework, students’ perceptions of teacher expectations influence their academic engagement through processes involving intentional adjustments to their self-schemata and expectancies for success. One key process that warrants further exploration is intentional self-regulation, which may serve as a crucial mediating mechanism in this relationship.

### 4.2 The mediating role of intentional self-regulation

Through longitudinal mediation analysis, this study confirms Hypothesis 2 by revealing how middle school students’ perceptions of teacher expectations affect their academic engagement through intentional self-regulation. The results extend our understanding of the situated expectancy-value theory (Wigfield and Eccles, 2000; Eccles and Wigfield, 2020) by demonstrating the pivotal mediating role of intentional self-regulation in this process.

Intentional self-regulation, as an active regulatory process, can be elucidated through the progression from “general self-schemata” to “expectations of success” within the expectancy-value theory model, fundamentally embodying the core characteristics of intentional self-regulation. Specifically, when students perceive high expectations from teachers, they adjust their self-schemata, including self-concept and beliefs about their own abilities. This process entails re-evaluating and reorganizing self-related information, which is central to intentional self-regulation. Through this self-regulation, students establish new learning goals, modify success expectations, and ultimately influence their learning behaviors and engagement. This goal-oriented regulatory mechanism enables individuals to reassess their abilities and objectives when perceiving others’ beliefs, and to adopt corresponding adaptive behaviors, thereby enhancing learning engagement (Gestsdóttir and Lerner, 2008; Eccles and Wigfield, 2020; Li et al., 2022). Concurrently, intentional self-regulation, through Selection, Optimization, and Compensation (SOC) strategies (Freund and Baltes, 2002), inherently involves students adjusting their success expectations and aligning these expectations with their perception of teacher expectations and personal goals. Students with high levels of intentional self-regulation can adeptly align their academic goals with perceived teacher expectations and continuously adopt adaptive learning strategies to overcome challenges (Gestsdóttir et al., 2009). They possess the ability to reevaluate their learning strategies and adjust their behaviors, transforming perceived teacher expectations into a catalyst for academic engagement (Cherng and Liu, 2017; Vattøy, 2020; Wahman and Anderson, 2021).

Through intentional self-regulation, students not only internalize their perceptions of teacher expectations but also actively monitor and regulate their learning processes. This proactive self-regulatory process facilitates the transformation of external teacher expectations into intrinsic learning motivation, thereby promoting increased levels of academic engagement (Bowers et al., 2016; Rubie-Davies et al., 2020).

Our research extends the application of situated expectancy-value theory by introducing intentional self-regulation as a mediating variable in understanding the impact of perceived teacher expectations on student learning engagement. The longitudinal mediation analysis reveals a partial mediating effect of self-regulation in the relationship between students’ perceptions of teacher expectations and their learning engagement. This finding suggests that intentional self-regulation play a role in how students’ perception of teacher expectations influence their academic engagement. While this partially supports the theoretical pathway from external influences to internal cognition and subsequent behavioral changes, it also indicates that other factors may be involved. The results contribute to our understanding of the complex dynamics in student learning processes, highlighting the interplay between perceived external expectations and internal regulatory mechanisms (Wigfield and Eccles, 2000; Han et al., 2022).

### 4.3 Limitations and future directions

This study has further validated and deepened the investigation of its thematic focus. However, several limitations remain to be addressed. Firstly, given that the study’s subjects were Chinese middle school students, it focused on exploring how students’ perceptions of homeroom teacher expectations influence their overall academic engagement. Future research could delve into a more detailed analysis of how students’ perceptions of expectations from specific subject teachers affect their academic engagement in those respective subjects. Secondly, the sample did not include ninth-grade students due to the structure of the middle school system. Subsequent research could investigate whether the perceptions of middle school teachers’ expectations have a lasting impact as students transition into high school. Thirdly, this study primarily focused on students’ perceptions. While this approach provided valuable insights, incorporating teachers’ perspectives could offer a more comprehensive view of the dynamics at play. Future research might benefit from integrating both student and teacher viewpoints to explore the Pygmalion effect more thoroughly. Lastly, the study focused on the effects of teacher factors. However, family dynamics, peer interactions, and academic achievements may also shape student academic engagement (Chase et al., 2014; Harris et al., 2020; McKellar and Wang, 2023). Future research should take a more holistic view of these external environmental factors, exploring how they interact with intentional self-regulation and their consequent effect on the academic development of middle school students.

## 5 Conclusion

This research enhances our comprehension of students’ perceptions of teacher expectations effect by exploring its influence on academic engagement and uncovering the underlying mechanisms. Employing a longitudinal design grounded in a robust methodological approach and well-developed theoretical context, the study explores the dynamics between students’ perceptions of teacher expectations and academic engagement among Chinese middle school students, emphasizing the mediating role of intentional self-regulation. Through cross-lagged panel analysis, the study revealed that (a) Middle school students’ perceptions of teacher expectations have a positive effect on student academic engagement, confirming their role as a significant factor in sculpting student academic engagement. (b) Middle school students’ perceptions of teacher expectations can positively predict students’ intentional self-regulation over time, which subsequently positively affects their academic engagement. (c) The mediation analysis further verified that intentional self-regulation serves as a significant longitudinal mediator in the relationship between middle school students’ perceptions of teacher expectations and academic engagement. These findings not only underscore the pivotal influence of students’ perceptions of teacher expectations on educational outcomes but also highlight the role of intentional self-regulation as a key intrinsic mechanism through which perceived teacher expectations impact middle school students’ academic engagement.

The implications of these findings advocate for a shift from traditional, logic-based teaching methods to a more emotion-centered approach. By tailoring expectations and instructional strategies to students’ needs and implementing diverse assessment methods, teachers can enhance students’ perception of support and understanding, thereby motivating students’ interest and determination in learning. This approach encourages students to embrace challenges and sustain a positive engagement with their studies.

Furthermore, it is imperative for students, schools, and families to develop strategies for intentional self-regulation. This involves guiding students in accurately evaluating their needs against available resources, setting realistic goals, and employing effective strategies to achieve these objectives, which in turn promotes optimal personal development.

## Data Availability

The datasets presented in this article are not readily available because the data presented in this study are available on request from the corresponding author. These data are not publicly available because they are part of an ongoing study, which requires maintaining confidentiality and integrity to ensure the validity of future, more in-depth research findings. Requests to access the datasets should be directed to wanghy178@nenu.edu.cn.
